# Seeing the Axial Line: Evidence from Wayfinding Experiments

**DOI:** 10.3390/bs4030167

**Published:** 2014-07-03

**Authors:** Beatrix Emo

**Affiliations:** Bartlett School of Graduate Studies, University College London, 1-14 Woburn Place, WC1H 0NN London, UK; E-Mail: b.emo@ucl.ac.uk; Tel.: +44-207-679-2000

**Keywords:** wayfinding, space syntax, eye tracking, real-world, spatial configuration, spatial geometry

## Abstract

Space-geometric measures are proposed to explain the location of fixations during wayfinding. Results from an eye tracking study based on real-world stimuli are analysed; the gaze bias shows that attention is paid to structural elements in the built environment. Three space-geometric measures are used to explain the data: sky area, floor area and longest line of sight. Together with the finding that participants choose the more connected street, a relationship is proposed between the individual cognitive processes that occur during wayfinding, relative street connectivity measured through space syntactic techniques and the spatial geometry of the environment. The paper adopts an egocentric approach to gain a greater understanding on how individuals process the axial map.

## 1. Introduction

Space syntax analyses of urban environments offer a successful way of examining aggregate pedestrian and vehicular movement. Whilst space syntax techniques do not, in themselves, account for individual motivation, the hypothesis has been proposed that the variables upon which space syntax techniques are based are the same as those used by individuals [1]. This paper offers initial experimental evidence in support of this hypothesis, for the case of pedestrian navigation in urban environments.

The paper examines findings from an eye tracking study based on real-world stimuli; behavioural decisions and eye tracking data during wayfinding at city street corners are analysed. The location of the fixations is shown to be related to structural elements of the viewshed. Space-geometric measures are used to account for the gaze bias; the relevance of an egocentric approach is affirmed.

The paper begins with a discussion of relevant previous work on spatial decision-making and the impact of structural properties of the environment. An overview of the data collection methods and the main results is given. The main body of the paper examines the location of the fixations; the role of the spatial geometry of the scene is highlighted. The paper ends with a discussion of the possible implications of the findings and identifies avenues for future research.

### 1.1. The Role of Spatial Configuration on Wayfinding

Wayfinding is not purely random; it follows psychological patterns based on visual perception. It can be defined as the decision-making process stage of navigation, where navigation is composed of locomotion and wayfinding [2]; wayfinding is necessarily related to choices made by the individual. Several factors are known to affect wayfinding behaviour; environmental variables are especially interesting, because they are derived from the environment itself (and not from the individual or from actions). Structural information is a type of environmental variable that refers to how building blocks are positioned in relation to the street. Such information is particularly relevant when someone is navigating to somewhere they have not been before. The relevance of structural information for human navigation was first highlighted by Lynch in his seminal study [3], in which he identified five elements of the built environment that were present in mental maps. Weisman’s paper directly identified spatial configuration as a relevant factor during wayfinding [4]. The proposition resulting from these early research initiatives is that, during wayfinding, humans draw on information on how paths in a city are linked; this notion is called spatial configuration.

Spatial configuration is defined as the way the relationship between any two spaces is altered by their connection to a third space (following the definition of [5]). Space syntax techniques propose a way of measuring spatial configuration [5,6]; they have been used to explain how the structure of a space determines how it is used. The first substantial study to test the role of spatial configuration on individual behaviour did so in a number of virtual environments [7]; it showed that patterns in pause behaviour are related to properties of the environment. A few papers have examined the role of spatial configuration in real situations; an influence of spatial configuration on wayfinding performance [8] was substantiated in a later study that examined a large number of participants during wayfinding in hospitals [9]. Measures of spatial configuration are also used to explain the difference in the wayfinding performance of novices compared to experienced users of an (existing) complex, multi-level building [10]. To date, much less is known about the role of spatial configuration on individuals compared to its effect on aggregate pedestrian movement, and any work at the individual level has not been tested in real-world environments: this is one of the aims of the paper.

### 1.2. Measuring Spatial Configuration through Space Syntax Analysis

Space syntax is a set of theories and techniques that examines how we interact with the environment. As a tool, it offers a way of measuring spatial configuration that has proven useful for architects and urban planners. Space syntax analyses of urban environments use a representation of the street grid based on axial lines. Axial lines are defined as the longest and fewest lines of sight with the potential for movement completing the network. The ensuing network is analysed as a graph, with the streets as nodes and the connections as the lines between the nodes. Initial space syntax methods were based on axial lines, which led to graph measures constructed around the topological properties of the grid (“axial map”). A refinement of the analysis weights the graph according to the angular displacement between street segments [11,12,13]; for segment angular analysis, axial lines are broken down at each junction into segments, so that each segment begins and ends at an intersection with another line. Two centrality-based measures of graph theory [14], closeness and betweenness, are often used in space syntax analysis; they correspond to different types of behaviour. Integration, a measure of closeness, reflects how likely it is that a segment is an origin or destination segment. It represents the centrality of a street in relation to the network as a whole. Choice, a measure of betweenness, reflects the likelihood that a segment is an intervening space in between an origin and a destination. It refers to the number of streets that are passed through on a journey. Taken together, measures of integration and choice are useful for analysing the wayfinding behaviour of individuals and will be used in the ensuing analysis.

### 1.3. Using Space-Geometric Measures to Assess the Role of Spatial Configuration on Wayfinding

A challenge in applying space syntax measures of spatial configuration to the behaviour of individuals lies in the allocentric nature of space syntax measures; this has been addressed to some extent in viewshed analysis. Viewsheds refer to what can be seen from a particular standpoint; a relevant type of viewshed for architectural research is isovists, which are 2D polygons, taken at a certain height, that represent the visible area from generating locations [15,16]. Viewshed analyses are relevant for an egocentric examination of how individuals behave in the built environment [7,17,18,19,20]; a link between viewshed properties and human behaviour has been proposed [21,22,23]. An extension of such an approach draws on the spatial geometry of the scene as perceived by the individual [24,25]. Such a form of analysis is based on what can actually be seen, as opposed to what could be visible from a theoretical point of view, and can be tested by examining eye movements. It is, arguably, a more relevant way to address the role of spatial configuration on the behaviour of individuals and provides a way of testing the notion that depth of view is held to be especially relevant for individual spatial decision-making [26,27]. Three space-geometric measures are introduced in this paper: floor area, longest line of sight and sky area.

### 1.4. Recording Eye Movements during Wayfinding

The benefit of collecting eye movement data for this study is the ability to record where participants direct their visual attention using non-verbal methods. Eye movements are analysed to determine fixations, which are periods of relative stability during which information is gathered. To date, only a few studies have examined gaze bias during individual spatial decision-making. One such study records the location of fixations during path search and path recall tasks, using artificially-created stimuli; the gaze bias patterns of participants performing the path search task suggests that attention is the spatial geometry of the scene [25].

Two models of visual attention are accounted for in this study. The bottom-up approach suggests that visual attention is based on stimulus-related properties [28]; it is accounted for through the variation of lighting conditions in the stimulus set. The top-down approach is centred on task-related influences; it suggests that viewing patterns differ according to different tasks [29] and is accounted for in this study by comparing the viewing patterns of spatial and non-spatial tasks.

## 2. Method

### 2.1. Experimental Procedure

Fifteen participants view photographs of street corners and choose which way to go. Eye tracking data is recorded using a desktop-based ASL EyeTrac 6000 pan/tilt optics remote eye tracker.

**Figure 1:**
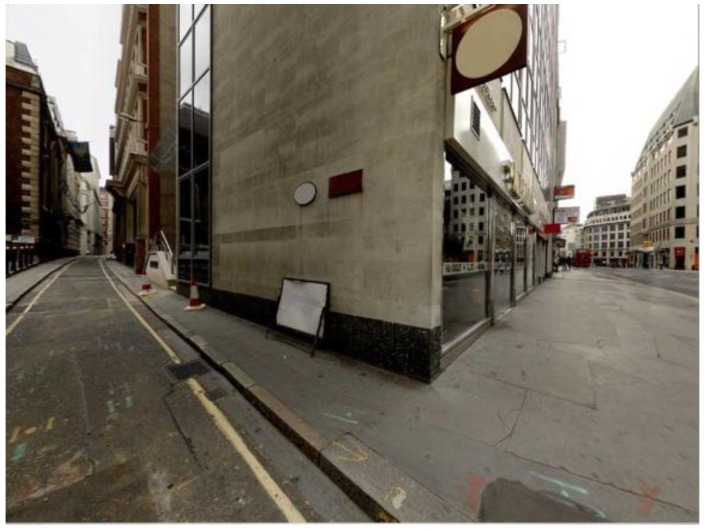
Example stimulus.

The stimuli are 28 photographs taken at urban street corners in the City of London and taken specifically for the study (see Figure 1). This particular area of London, the historical centre of the city, is chosen because of its specific spatial quality; whilst it developed in an unplanned “organic” fashion, it retains an internal logic, whereby one is never more than two streets away from a larger road [5]. Each stimulus presents a decision point with a distinct binary choice of one left and one right path alternative. A number of criteria are used to determine the location and specific angle of each photograph (refer to [30] for more detail); overall, the stimuli aim to present the viewer with a clear understanding of the spatial geometry of the environment. The final stimulus set includes a version of each stimulus that is mirrored on the vertical axis to test for any left/right bias.

Some of the factors known to affect wayfinding behaviour are controlled for in the stimulus set. The influence of familiarity [10] is controlled for, by asking participants post experiment whether they recognized any of the locations; none of the participants recognized the locations. The presence of attractors affects wayfinding behaviour [31]; in this study, it is accounted for by including stimuli in which (i) people; (ii) vehicles and (iii) shops are present in separate instances. A limitation of the study is that individual differences are not specifically accounted for [32]: these should be addressed in future research.

Participants respond to two spatial tasks. The undirected spatial task relates to the most basic form of wayfinding activity. Participants are asked “which way would you go?” with no other information being provided. The directed spatial task specifically sets out to test the role of street connectivity on wayfinding behaviour. Participants are asked “which way would you go to find a taxi rank?”

Task-related viewing behaviour is controlled for by including a non-spatial task, namely a recall task. Ten stimuli are used in the recall task, five from the original set and five supplementary images; participants indicate whether a stimulus has already been shown in the experiment or not. Stimulus-derived viewing behaviour is tested through the variation of lighting conditions; two stimuli, in which one path alternative is brighter than the other, are digitally manipulated, so that both path alternatives seem equally bright.

Each participant is shown the full set of stimuli, including the mirrored versions, in random order. The spatial tasks are blocked, with the undirected instance being shown first; this is the necessary procedure to avoid participants responding to the more specific directed task in the undirected instance. A white screen with a central black cross is shown for 2 s in between all slides (stimuli and instruction slides) to foster similar viewing behaviour for each stimulus. Stimuli remain on screen until a response is made. Participants view half the stimuli when responding to the undirected spatial task; following on-screen instructions, participants view the remaining half of the stimuli while responding to the directed task. Subsequently, on-screen instructions ask participants to recall whether they have already been shown any of the following stimuli. Five randomly chosen stimuli from the full stimulus set are interwoven with the same number of images not previously viewed; these images are then shown in random order. A nine-point calibration grid is used to calibrate the eye tracker before and after data collection.

### 2.2. Analytic Methods

The behavioural data is tested for a number of different factors. First, any left/right bias in the decisions is tested by recording whether choices made for the non-mirrored stimuli match those made in the mirrored conditions. The total number of decisions that match per participant are tested against a random model in which half go one way, using a one-sample *t*-test.

**Figure 2:**
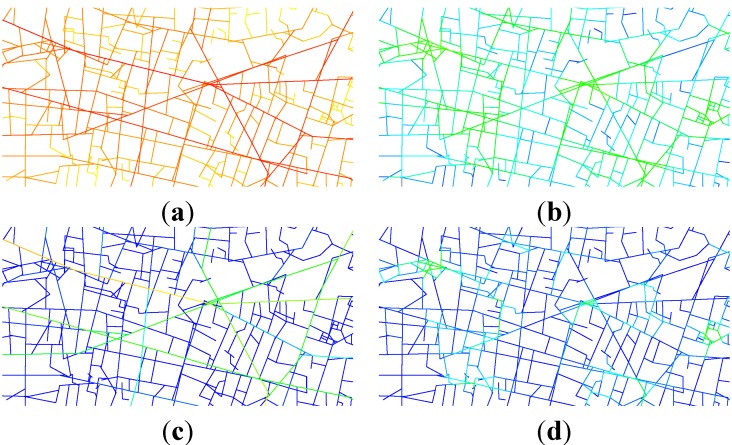
Segment angular maps of the centre of London. (**a**) Global integration. (**b**) Local integration. (**c**) Global choice. (**d**) Local choice. The maps are coloured from red (representing a more integrated space) to blue (a more segregated space).

Behavioural decisions are also assessed according to whether or not they follow the more connected path (refer to [30,33]). The more connected path is the one that is more integrated according to one of four space syntax measures: integration radius n; integration radius 100 m; choice radius n; and choice radius 100 m. A segment angular model of the City of London is used, with a catchment area of three miles to avoid any edge effects (the graphs from which the measures are recorded are illustrated in Figure 2; the difference between the measures can be seen from the different colouring of the graphs). The measures are recorded using Depthmap software [34]. For each stimulus, the total number of decisions per participant that can be modelled according to the spatial configuration is recorded. The results are tested against a random 50:50 model using a one-sample *t*-test.

Further analysis compares the bias towards the more connected street in the undirected and directed spatial tasks; the data is presented as a percentage change, so no statistical test of significance is given. The influence of the presence of attractors is tested by assessing whether or not responses are skewed towards attractors in those stimuli where attractors are present. Finally, bias relating to lighting conditions is tested: the total number of decisions per participant that are unaltered despite changes in light conditions are compared to a random 50:50 model. The level of significance for the attractor and lighting controls are tested using a one sample *t*-test, although the effect of the tests is limited given the very low sample sizes.

Two forms of analysis are applied to the eye tracking data. Fixations are defined as having a minimum time of 0.1 s; the initial fixation is discarded in all cases where it is directed at the centre of the stimulus, as this is likely to be dictated by the location of the crosshair in the disambiguating screen shown in between stimuli. First, the general pattern of the fixations is examined through: (i) the number and duration of fixations; (ii) the location of the initial and final fixations; (iii) how often the centre line is crossed; and (iv) time spent in the eventually chosen half of the stimulus. Any left/right bias and bias towards the eventually chosen path are tested against a random 50:50 model using a one-sample *t*-test.

Second, the location of fixations is analysed. Fixation density graphs, in which each axis is split into 30 bins, are used to explain the data. The distribution of fixations along the x-axis is tested for bias towards the longest line of sight. The x-axis bin of the longest line of sight is identified; the total number of fixations for all participants directed at (i) the longest line of sight and (ii) the bins immediately on either side of the longest line of sight are expressed as a proportion of the total number of fixations per path alternative. The distribution of fixations along the y-axis is tested for bias towards the horizon and sky lines. The y-axis bins of the horizon and sky lines are identified; the total number of fixations for all participants directed at, and between, the horizon and sky lines are expressed as a proportion of the total number of fixations per stimulus. Differences in the distribution of fixation patterns between the undirected and directed spatial tasks are tested using a Kolmogorov–Smirnov test. The gaze bias due to the presence of attractors is tested by identifying the average number of fixations per participant that followed attractors. The influence of lighting conditions on viewing behaviour is tested by examining the average increase of fixations towards the path alternative where the lighting conditions have been altered, compared to the unaltered state. As there is no expected value for the number of fixations in the controls for lighting and attractors, no statistical test of significance is given.

The primary aim of the recall task is to test the difference in the viewing pattern between the spatial tasks compared to the recall task; a Kolmogorov–Smirnov test is used to measure the similarity of the distributions along the x-axis.

## 3. Results

### 3.1. Behavioural Data

The behavioural data shows that the decisions are not random. The majority of decisions, 82.38% (*p* < 0.01), are consistent, even when the stimulus is mirrored on the vertical axis. Thus, the majority of subjects choose the same path regardless of whether it is shown as the left-hand or right-hand path choice. The average response time is 2.61 s (range: 0.694–19.01); the average response time for the undirected spatial task is greater than for the directed spatial task (3.0 s *vs*. 2.21 s, respectively).

Results show that two thirds of all decisions made are towards the more connected street (see Table 1). The measure of spatial configuration for which this is highest is global integration (76.90%, *p* < 0.01); this is followed by local integration at 71.19% (*p* < 0.01). The fact that both integration measures show higher correlations than both choice measures suggests that there is something about the way that integration reports centrality within a network that corresponds to individual spatial-decisions. Global choice also shows a strong correlation (70.24%, *p* < 0.01); however, a reduced number of decisions (54.05%, *p* < 0.02) follow the space syntax measure of local choice.

**Table 1:** Decisions made independent of task.

	Integration r = n	Integration r = 100 m	Choice r = n	Choice r = 100 m
No. of decisions	21.53	19.93	19.67	15.14
%	76.90	71.19	70.24	54.05
*p*-value	<0.01	<0.01	<0.01	<0.02

Findings also show that more decisions follow measures of spatial configuration in the directed search task than in the undirected search task, especially for global measures of spatial configuration; the average number of decisions per participant that favour the more connected street increases for global integration by 2.87 times (equivalent to a 30% increase) and for global choice by 2.2 times (equivalent to a 25% increase). In addition, decisions strongly favour attractors where these are present (91.67%, *p* < 0.01 of decisions follow attractors); this finding is in line with previous research. No significant effect of lighting conditions on participants’ decisions is found; 88.33%, *p* < 0.01 of decisions are not affected by the light conditions. The eye tracking data allows these findings to be analysed in greater detail.

### 3.2. Eye Tracking Data

#### 3.2.1. Time Course Pattern

For the spatial tasks, there are, on average, 4.20 fixations (SD = 1.95) per participant per stimulus, lasting 0.34 s (SD = 0.04 s). Each participant crosses the centre line on average 1.65 times (SD = 0.73). There is a significant tendency to look left first; on average, each participant looks left first in 70% of cases (*p* < 0.01). This is coupled with a tendency to place the final fixation towards the eventually chosen path; this occurs in 70% (*p* < 0.01) of cases. More attention is directed towards the chosen path; on average, each participant spends 12.0 s compared to 9.07 s at the eventually chosen path alternative. The average viewing behaviour for each stimulus during the spatial tasks can thus be described as looking left initially, crossing the centre line almost twice per stimulus and viewing the eventually chosen path last, having spent more time in the eventually chosen half.

A different time-course pattern is present in the recall task. Each participant on average makes fewer fixations (2.26, SD = 0.86), for shorter duration (0.29 s), and the number of times they cross the centre line is almost half at 0.89 times. These results suggest that the nature of the task (spatial *vs*. non-spatial) affects viewing patterns.

#### 3.2.2. Location of Fixations

**Figure 3:**
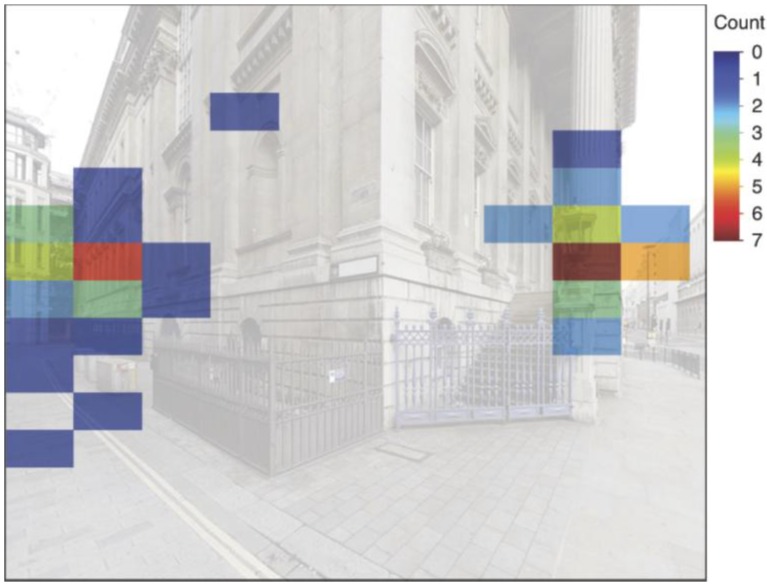
Example distribution of fixations of all participants for one stimulus.

The gaze bias is not random. Across all stimuli, fixations are concentrated at two areas of interest (see Figure 3).

**Figure 4:**
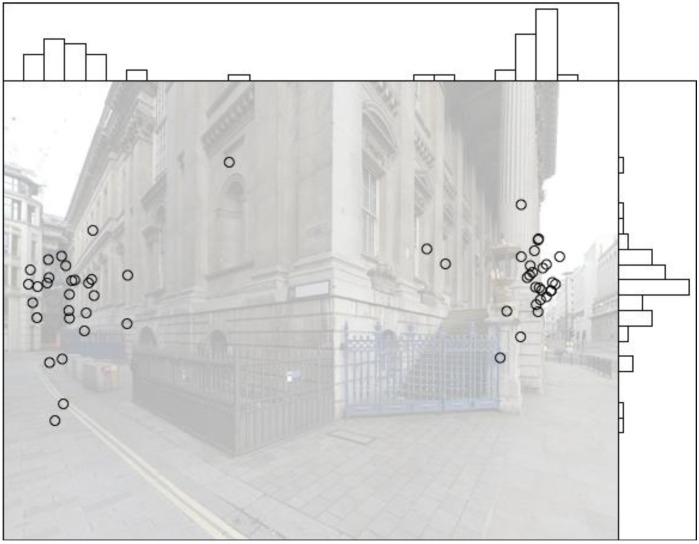
Example fixation data of all participants for one stimulus, with accompanying fixation density graphs.

These areas of interest appear consistently across all stimuli and loosely correspond to the two path alternatives in each stimulus. The distribution of fixations along the x- and y-axes is easily illustrated through fixation density graphs (Figure 4).

The distribution of fixations along both axes is tested for bias (i) towards the longest line of sight on the x-axis and (ii) between horizon and sky lines on the y-axis. Findings show that, on average, 18% of fixations per path alternative are directed specifically at the longest line of sight and 74% at the longest line of sight, including the bins immediately on each side. Thus, the distribution of the majority of fixations on the x-axis can be explained through the longest line of sight. On the y-axis, fixations tend to lie in between the horizon and sky lines; on average, 78% of fixations per stimulus are directed at this region. This finding is in keeping with results from a previous study [25], as well as with the knowledge based on the universal viewing behaviour of photographs.

**Figure 5:**
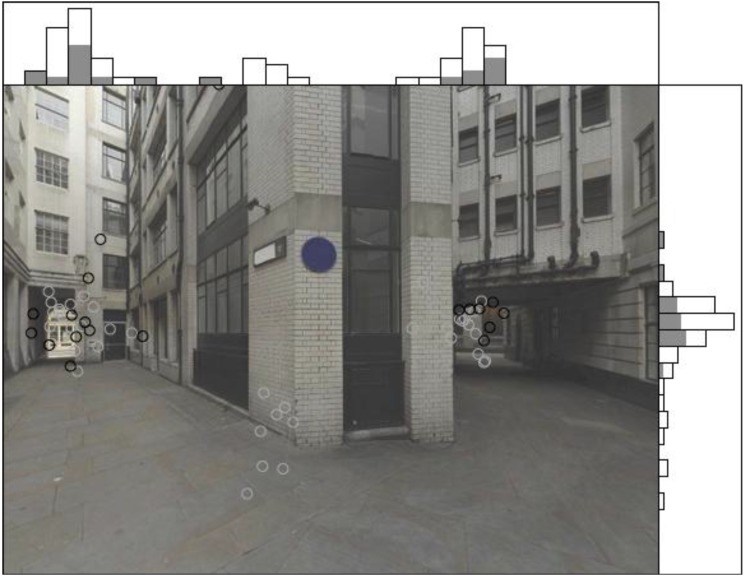
Comparison of the fixation data between the undirected (white) and directed spatial tasks (black fixation and bars), showing data from all participants for one stimulus.

A small difference in the viewing patterns between the undirected and directed spatial tasks is found (see Figure 5). A Kolmogorov–Smirnov test on the two distributions shows D = 0.1, *p* < 0.01, where D is the maximum difference between the distributions. Taken together, the viewing patterns and behavioural decisions confirm that there is an effect of the directed compared to the undirected spatial task. The gaze bias is affected by the presence of attractors; on average, 73.51% of fixations in the stimuli where attractors are present are directed towards them. These findings confirm the results from the behavioural decisions that attractors affect wayfinding behaviour. Furthermore, no strong changes in gaze bias are detected following changes in lighting conditions; on average, a slight increase towards the altered state is detected (11.31%). Therefore, in conjunction with the behavioural decisions, there is some evidence to support the view that the distribution of eye movements is not stimulus-derived.

**Figure 6:**
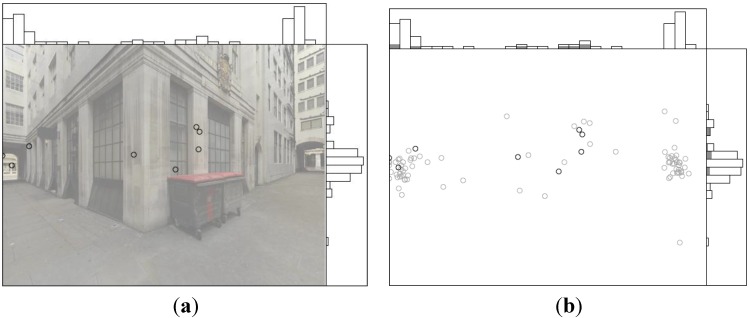
Comparison of fixation data between the recall and spatial tasks, showing the data from all participants for one stimulus. (**a**) Recall task fixations only. (**b**) Recall task data (black dots and bars) superimposed on the spatial task data (white dots and bars).

The main difference in the gaze bias patterns during the recall task compared to the spatial tasks is the scarcity of fixations (see Figure 6). A difference is found between the overall shape of the two distributions; a Kolmogorov–Smirnov test shows D = 0.11, *p* < 0.02. Only tentative conclusions can be reached regarding the difference in the distributions for individual stimuli; this is due to the very low number of fixations during the recall task. No significant differences are found when looking at each stimulus individually, except for one stimulus (depicted, D = 0.48, *p* < 0.05); future work should test this further (e.g., for non-spatial tasks where the average number of fixations per stimulus is higher).

## 4. Discussion

The paper tests the relevance of space syntax measures of spatial configuration on decisions made by individuals during wayfinding. It reports the results of an eye tracking study in which participants respond to basic wayfinding tasks; controls account for stimulus-derived and task-relating viewing patterns. The use of real-world stimuli gives the study the ecological validity that virtual-world studies may lack. The fixation data sheds greater light on the behavioural decisions and allows for a detailed analysis of the location of fixations in relation to the spatial structure of the scene.

Findings show that there is a tendency to choose the more connected street [30,33]; two thirds of decisions select the more connected street, analysed using integration and choice at local and global scales. Greater correlations with the wayfinding decisions are found with integration, which reflects the centrality of the street within the network, suggesting that participants somehow read the centrality of the street from the current standpoint; the fixation data allows for this phenomenon to be examined in greater detail.

The eye movement data shows that attention is paid to the structural information in the stimuli. For each stimulus, there are two areas of interest, corresponding to the two path alternatives. On the vertical axis, fixations are focused in between the horizon and sky lines; on the horizontal axis, fixations are clustered around the longest lines of sight. Thus, participants seem to be “reading” information on how each path alternative relates to the global network from an understanding of the local spatial structure.

**Figure 7:**
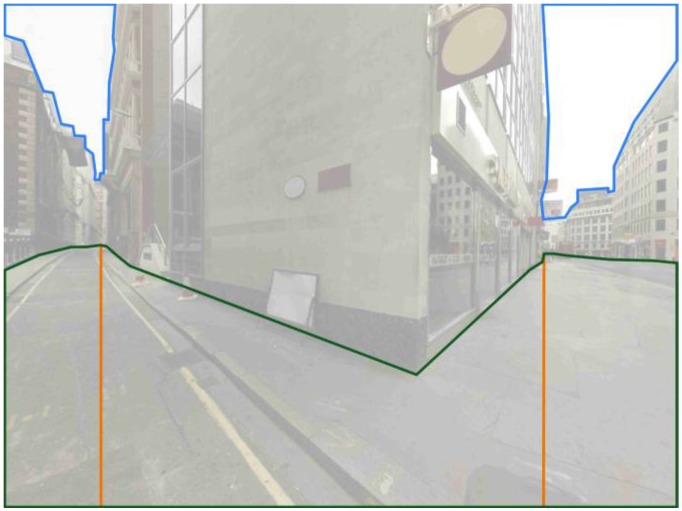
Three space-geometric measures: floor area (outlined in green), longest line of sight (orange) and sky area (blue).

An explanation for the distribution of fixations across both axes is proposed that draws on the spatial geometry of the scene; the approach is a development of that used in [24]. Three space-geometric measures are used: sky area, floor area and longest line of sight (see Figure 7). The floor area refers to the amount of walkable surface visible from the current standpoint; it is used to define the horizon line for each path alternative. The longest line of sight is the maximum of the floor line for each path alternative. The sky area refers to the amount of visible sky and is used to define the sky line. This is a data-driven approach and constitutes a first step in assessing the role of space-geometric features in the prediction of human gaze patterns during wayfinding. The benefit of using these three measures is to be able to explain the distribution of fixations across both the horizontal and vertical plane; the fixation data shows that the longest line of sight is critical along the x-axis, and that the horizon and sky lines are relevant along the y-axis. Previous research has highlighted the importance of depth of view for wayfinding [25,27] and the relevance of lines of sight for space syntax analysis [5,26], and thus, a key space-geometric element is the longest line of sight; however, in contrast to previous studies (e.g., [25]), the approach here accounts for the horizontal and vertical distribution of areas of interest in relation to the spatial structure of the scene.

## 5. Conclusions

The axial line has been shown to be a powerful form of representation and one that may well be reflected in individual spatial decision-making [1]. The findings in this paper open up the intriguing possibility of understanding how we process information relating to spatial configuration; depth of view seems to be a critical factor. The results suggest a relationship between individual spatial decision-making, relative street connectivity and the spatial geometry of the built environment. People choose the more connected street and, when making that decision, pay attention to the spatial geometry of the built environment. Given that participants also favour the more connected street, a tentative link is proposed between space-geometric measures and spatial configuration. Future work should account for additional compounding factors, such as, for example, the role of individual differences. Additional work is needed to link the findings of this paper to how the physical human experience relates to the axial map and relates to the mental image [35,36].

This paper argues for an egocentric approach to address the role of spatial configuration on individual spatial decision-making. While space syntax measures are mostly allocentric (the value assigned to a street segment holds true for all possible locations and orientations along that street), egocentric methods allow these measures to be applied to decisions made by individuals. The adoption of an egocentric approach allows for a novel form of viewshed analysis that tests the role of spatial geometry on real-world wayfinding behaviour.
